# Lifting the veil on arid-to-hyperarid Antarctic soil microbiomes: a tale of two oases

**DOI:** 10.1186/s40168-020-00809-w

**Published:** 2020-03-16

**Authors:** Eden Zhang, Loïc M. Thibaut, Aleks Terauds, Mark Raven, Mark M. Tanaka, Josie van Dorst, Sin Yin Wong, Sally Crane, Belinda C. Ferrari

**Affiliations:** 1 School of Biotechnology and Biomolecular Sciences, University of New South Wales, Sydney, 2052 Australia; 2 Australian Antarctic Division, Department of Environment, Antarctic Conservation and Management, 203 Channel Highway, Kingston, TAS 7050 Australia; 3 Commonwealth Scientific and Industrial Research Organisation, Mineralogical Services, Urrbrae, SA 5064 Australia

**Keywords:** Antarctica, Soil Microbiome, Bacteria, Eukarya, Archaea, Conservation Ecology

## Abstract

**Background:**

Resident soil microbiota play key roles in sustaining the core ecosystem processes of terrestrial Antarctica, often involving unique taxa with novel functional traits. However, the full scope of biodiversity and the niche-neutral processes underlying these communities remain unclear. In this study, we combine multivariate analyses, co-occurrence networks and fitted species abundance distributions on an extensive set of bacterial, micro-eukaryote and archaeal amplicon sequencing data to unravel soil microbiome patterns of nine sites across two east Antarctic regions, the Vestfold Hills and Windmill Islands. To our knowledge, this is the first microbial biodiversity report on the hyperarid Vestfold Hills soil environment.

**Results:**

Our findings reveal distinct regional differences in phylogenetic composition, abundance and richness amongst microbial taxa. Actinobacteria dominated soils in both regions, yet Bacteroidetes were more abundant in the Vestfold Hills compared to the Windmill Islands, which contained a high abundance of novel phyla. However, intra-region comparisons demonstrate greater homogeneity of soil microbial communities and measured environmental parameters between sites at the Vestfold Hills. Community richness is largely driven by a variable suite of parameters but robust associations between co-existing members highlight potential interactions and sharing of niche space by diverse taxa from all three microbial domains of life examined. Overall, non-neutral processes appear to structure the polar soil microbiomes studied here, with niche partitioning being particularly strong for bacterial communities at the Windmill Islands. Eukaryotic and archaeal communities reveal weaker niche-driven signatures accompanied by multimodality, suggesting the emergence of neutrality.

**Conclusion:**

We provide new information on assemblage patterns, environmental drivers and non-random occurrences for Antarctic soil microbiomes, particularly the Vestfold Hills, where basic diversity, ecology and life history strategies of resident microbiota are largely unknown. Greater understanding of these basic ecological concepts is a pivotal step towards effective conservation management.

## Background

East Antarctica constitutes up to two-thirds of the continent and is home to some of the oldest, coldest and most oligotrophic soils on Earth [1]. Apart from ice-free patches along the coast, most of the sector is covered by a thick layer of permafrost [2]. The Windmill Islands, an ice-free region situated near the Australian Casey research station, consists of five major peninsulas and a number of rock-strewn islands. Approximately 100 km to the north lie the Vestfold Hills, a large expanse of low-lying hilly country deeply indented by sea-inlets and lakes [3, 4]. These diverse edaphic habitats are a legacy of age-involving varied geological and glaciological processes [5]. Soil microbial diversity and functional ecology of the hyperarid Vestfold Hills is virtually unexplored, whilst previous studies at the Windmill Islands have disclosed a relatively high proportion of novel bacterial phyla [6]. However, knowledge on archaea and micro-eukaryotes is still lacking [7]. This is in stark contrast to other regions such as the McMurdo Dry Valleys and Antarctic Peninsula [6, 8, 9]. In our understanding of soil microbiota across the different bioregions of terrestrial Antarctica, addressing these deficiencies will not only improve our understanding of Antarctic microbial biogeography but also guide future conservation planning strategies [10].

Climate and soil age abiotic factors such as pH, moisture and nutrient content exert a strong influence on Antarctic species distribution and life histories [1, 7, 11–13]. These properties may co-vary with local lithology, pedology and aspect—leading to a myriad of edaphic niches [14]. In turn, their microbial occupants are key to establishing and maintaining core ecosystem processes, occasionally involving unique taxa with novel functional traits such as unique biosurfactants and trace gas assimilation as a novel mode of primary production [5, 15]. It is thereby a widely accepted concept that the capacity of microbes to access and utilise resources, as well as tolerate different levels of stress, contributes significantly to the structuring of microbiota dwelling within these oligotrophic soils.

However, our ability to unravel these basic ecological concepts in cold regions has been limited by the small number and depth of studies available [16]. Moreover, the majority of relevant studies have largely been focused on bacteria only. Few micro-eukaryotic and archaeal-specific surveys have been reported on terrestrial Antarctic environments and so their ecological roles remain elusive [17–19]. All three microbial domains are likely to be responsible for the sustainability and evolution of the polar soil microbiome but contemporary dynamics will inevitably change due to the climate-driven emergence of new ice-free areas [2, 20–22]. As consequence, further clarification on their underlying drivers will establish a baseline from which to gauge ecological shifts, which is an important step towards effectively managing microbial biodiversity loss and conserving the key ecosystem functions offered by these assemblages [23–28, 29].

Projected twenty-first century expansion of ice-free habitats across eastern Antarctica means that tools for rapidly assessing soil ecosystem health, such as species abundance distributions (SADs), are gradually becoming more important in managing microbial biodiversity loss, especially in regions where survey data is scarce [2, 30]. In this study, we compiled bacterial 16S (*n* = 837), eukaryotic 18S (*n* = 162) and archaeal 16S (*n* = 144) rRNA gene amplicon sequencing data from soil samples spanning nine east Antarctic sites between the Vestfold Hills (*n* = 5) and Windmill Islands (*n* = 4). By taking a multivariate, exploratory network and modelling approach using fitted SADs we aim to (1) elucidate the previously unknown soil microbial biodiversity of the Vestfold Hills, (2) determine the driving processes (i.e. niche or neutral) underlying the microbial communities of east Antarctica and (3) clarify whether they differ between the Vestfold Hills and Windmill Islands.

## Results

### Amplicon sequencing yield and coverage

We recovered a total of 60,495,244 high-quality bacterial 16S rRNA gene sequences, which clustered into 36,251 operational taxonomic units (OTUs) at 97% identity cut-off. Our micro-eukaryotic and archaeal runs yielded a total of 1,299,519 18S rRNA and 13,373,072 16S rRNA gene sequences after read-quality filtering, which respectively clustered at 97% into 1511 and 589 OTUs (Table S1). Subsampled rarefaction curves of the pooled data revealed that bacterial, micro-eukaryotic and archaeal richness generally approached an asymptote at each site (Fig. S1).

### Comparative biodiversity of the east Antarctic polar soil microbiome

**Fig. 1:**
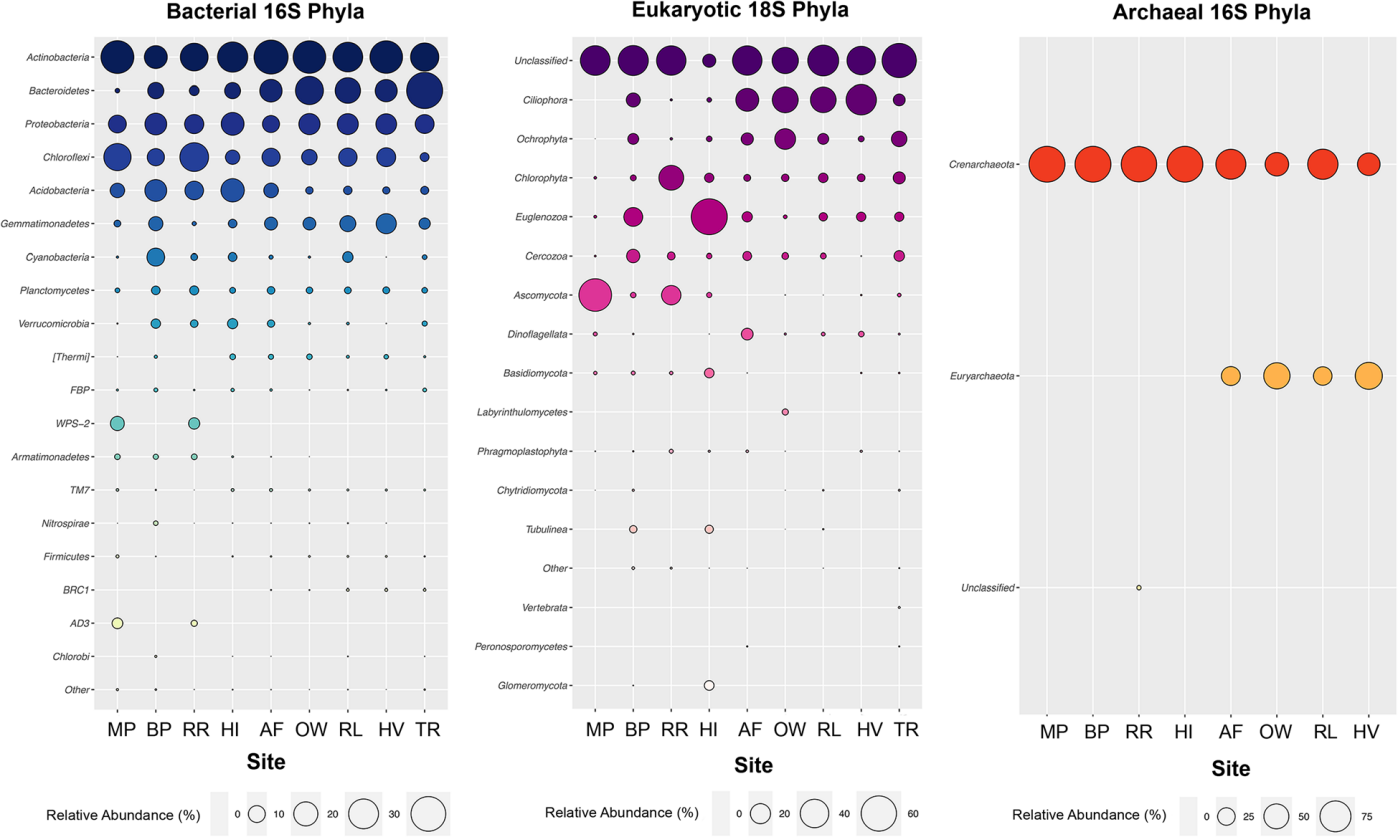
Bubble plots of relative abundance (%) per site of phyla-level composition of OTUs (97% cut-off), based on bacterial 16S (mean = 490 bp), eukaryotic 18S (mean = 125 bp) and archaeal 16S (mean = 470 bp) SSU rRNA sequences representing &gt; 0.001% of all normalised OTUs sorted by decreasing relative abundance. Greatest phylogenetic diversity is exhibited by bacteria followed by eukarya then archaea. Across all three domains, distribution of phyla abundances is generally uneven as a handful of taxa tend to dominate but strong compositional differences are apparent between the Windmill Islands and Vestfold Hills regions

At 97% identity, OTUs were classified into 63 bacterial, 27 micro-eukaryotic and three archaeal phyla. Distributions of phylum abundances for all three domains were uneven, as the majority of sites were dominated by a handful of taxa (Fig. 1). Soil bacterial communities predominantly consisted of the metabolically diverse *Actinobacteria* (30.5%) and *Proteobacteria* (14.6%). *Bacteroidetes* (24.9%) and *Gemmatimonadetes* (8.0%) were more prevalent at the Vestfold Hills, whereas *Chloroflexi* (17.8%) and *Acidobacteria* (13.6%) were present in greater relative abundances throughout the Windmill Islands. With the exception of Rookery Lake (RL = 4.2%), Browning Peninsula (BP = 10.9%) and Herring Island (HI = 3.1 %), *Cyanobacteria* abundance was relatively low across all sites. At Mitchell Peninsula (MP) and Robinson Ridge (RR), rare candidate phyla namely *Candidatus Eremiobacteraeota* (*WPS*-*2*) and *Candidatus Dormibacteraeota* (*AD3*) were present in higher relative abundances (&gt; 4.6%) compared to the other sites. At lower taxonomic levels, bacterial sequences classified into 169 classes, with members largely belonging to *Flavobacteria* (10.9%) and *Actinobacteria* (9.0%), followed by similar proportions (6.0%) of *Thermoleophilia*, *Chloracidobacteria*, *Gamma-proteobacteria* and *Alpha*-*proteobacteria* (Fig. S2).

For micro-eukaryotes, 18S rRNA gene sequences fell into six supergroups consisting of unclassified (46.9%), *Chromalveolata* (*Ciliophora* and *Dinoflagellata* = 20.6%), *Archaeplastida* (*Ochrophyta*, *Chlorophyta* and *Phragmoplastophyta* = 17.8%), *Excavata* (*Euglenozoa* = 5.4%), *Opisthokonta* (*Ascomycota*, *Basidiomycota*, *Labyrinthulomycetes*, *Chytridiomycota*, *Vertebrata*, *Peronosporomycetes* and *Glomermomycota* = 4.6%) and *Amoebozoa* (*Cercozoa* and *Tublinea* = 4.4%) (Fig. 1). Unclassified micro-eukarya remained dominant across all taxonomic levels, with moderately higher relative abundance observed throughout the Vestfold Hills (61.3%), compared with the Windmill Island sites (38.8%), particularly at The Ridge (TR). Fungal diversity contributed to a relatively small proportion (10.5%) of the total relative abundance for eukaryotic soil communities, except at MP and RR.

Archaeal diversity was predominantly distributed within the *Crenarchaeota* phylum (84.5%), whilst members of *Euryarchaeota* (15.0%) were mainly exclusive to the Vestfold Hills (Fig. 1). In addition, an unusually high proportion (2.3%) of unclassified archaea was observed at RR. At lower taxonomic levels, archaeal sequences belonged to six main families; *Nitrososphaeraceae* (84.5%) and *Halobacteriaceae* (15.0%), followed by unclassified, *SAGMA-X*, *Cenarchaeaceae* and *TMEG* families, collectively accounting for 0.01% of total relative archaeal abundance.

### Domain-level biotic interactions

**Fig. 2:**
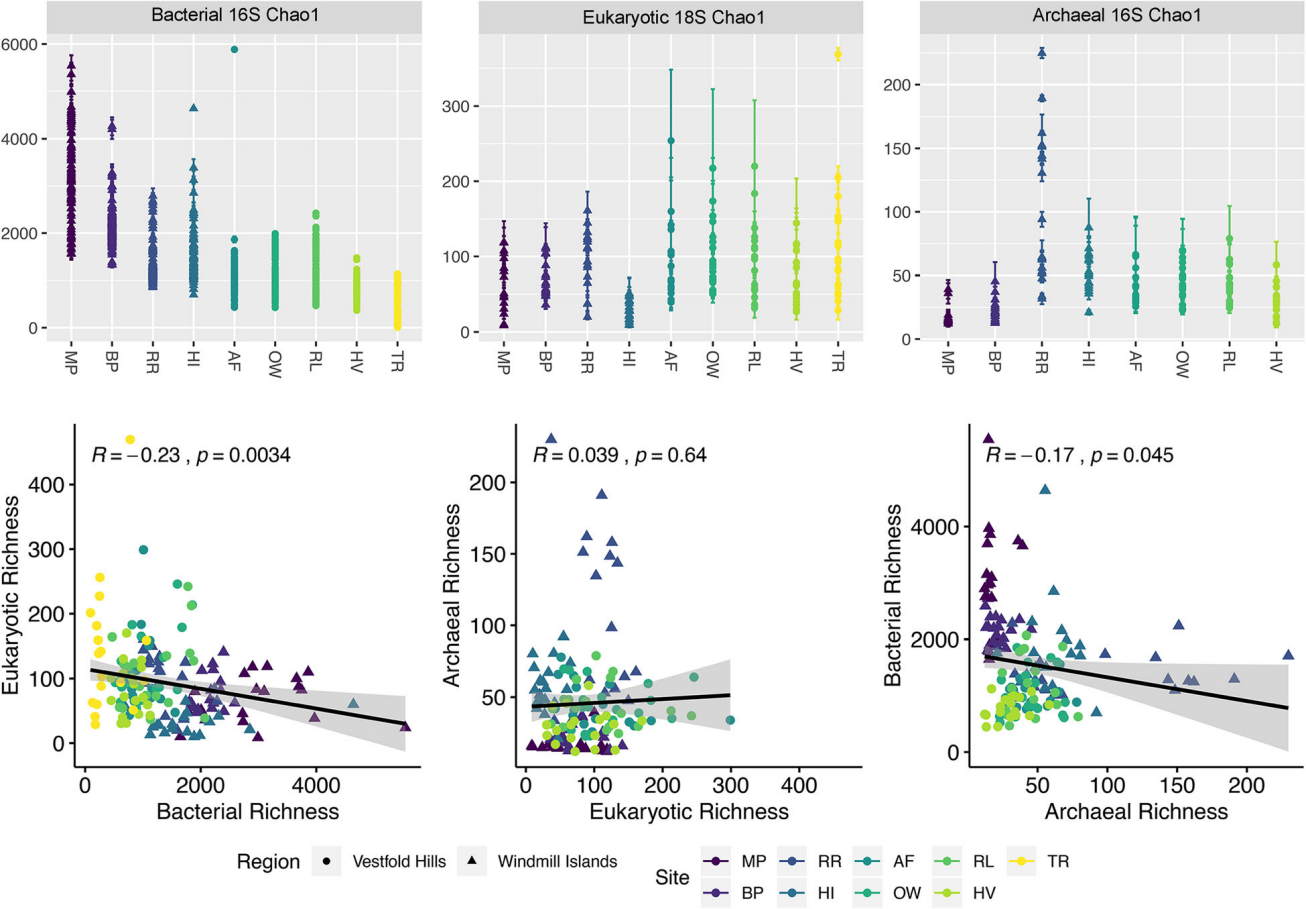
Chao1 richness estimates and correlations between our soil bacterial, eukaryotic and archaeal communities coloured by site. Polar soil bacterial communities demonstrated highest overall species richness estimates, particularly throughout the Windmill Islands region. Significant (*P* &lt; 0.05) negative correlations were detected between estimated bacterial species richness against the other two microbial domains

Non-metric multidimensional scaling (NMDS) ordination of microbial OTU communities and corresponding environmental metadata revealed that soils were conserved within sites and broadly by geographic region (Fig. S3). Apart from TR, sites at the Vestfold Hills were more homogenous in terms of community composition and measured soil parameters. Bacterial communities exhibited the greatest overall species richness based on Chao1 estimates (Fig. 2), particularly at the Windmill Islands (mean = 2270.1). In contrast, greater eukaryotic richness was observed throughout the Vestfold Hills (mean = 132.3). Archaeal communities exhibited the lowest overall species richness (mean = 50.9), with RR being an exception (mean = 106.4). Pearson’s correlations between domain-level pooled Chao1 richness estimates revealed weak but significant (α = 0.05) negative relationships of bacterial communities against both micro-eukaryotic (*R* = − 0.23, *P* = 0.0034) and archaeal (*R* = − 0.17, *P* = 0.045) communities. However, no significant correlation was found between micro-eukaryotic and archaeal richness (*R* = 0.039, *P* = 0.64).

**Fig. 3:**
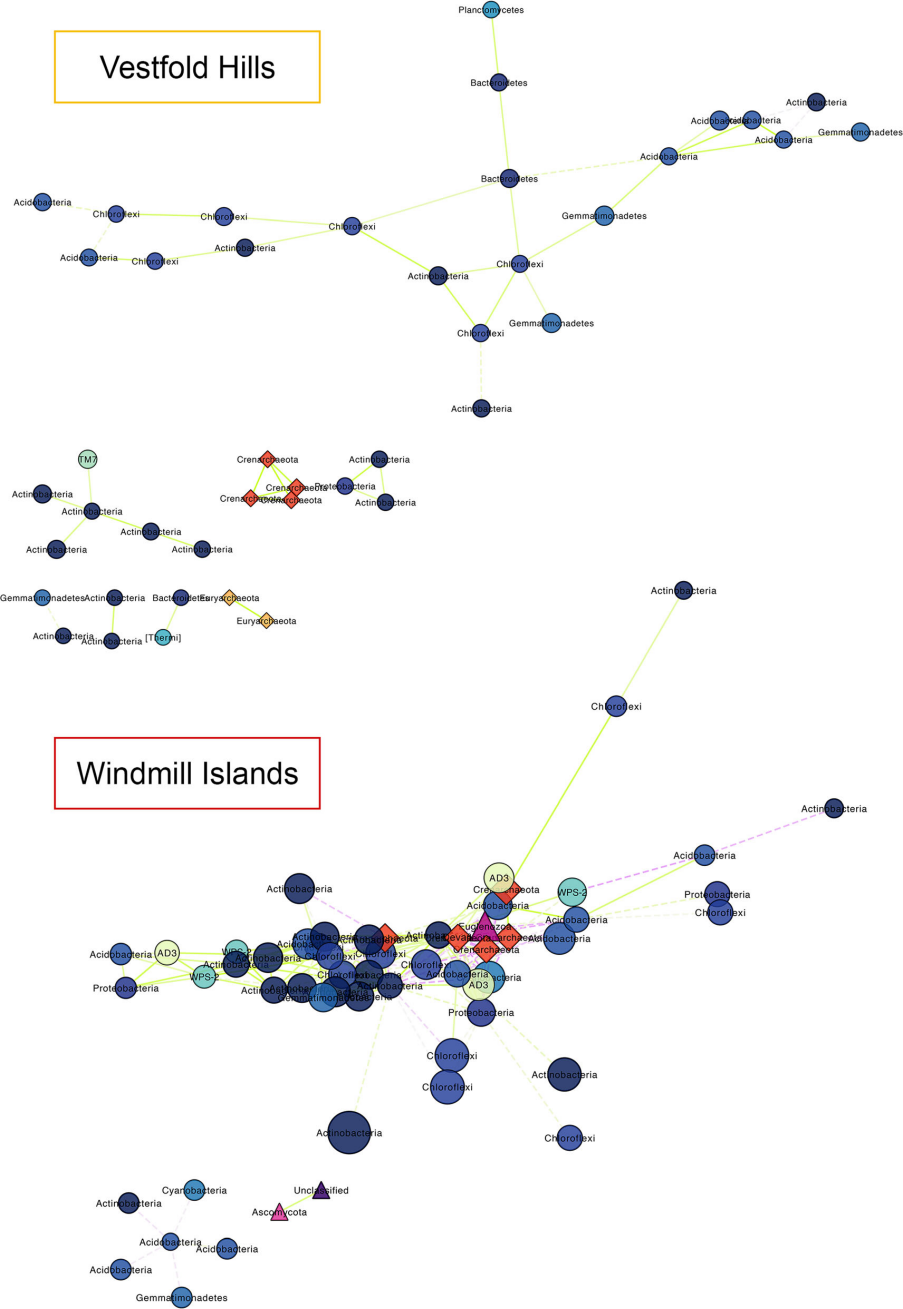
Domain-level OTU co-occurrence network of significant (*P* &lt; 0.001) and strongly correlated (*MIC* &gt; 0.8) OTU pairs between the Windmill Islands and Vestfold Hills. Nodes (circles = *bacteria*, triangles = *eukarya*, diamonds = *archaea*) and edges represent individual OTUs and their correlations respectively. Node size is proportional to their degree of connectivity and edge colour is based on linearity (green/solid = positive, purple/dashed = negative). Our soil microbial networks are comprised of moderately connected OTUs, more so at the Windmill Islands, structured amongst multiple components and forming a clustered topology. All three domains of life are present within the Windmill Islands network, most notably *Crenarchaeota* being strongly embedded and *Actinobacteria* forming the microbial backbone within these desert soils. In contrast, *eukarya* are absent from the Vestfold Hills network, suggesting possible competition

Domain-level networks displaying the co-occurrence of OTUs provided new insights on the potential sharing of niche spaces or interactions between co-existing taxa, many of which are understudied (Fig. 3). The resulting network for the Vestfold Hills consisted of 43 nodes (clustering coefficient = 0.2) and 44 edges (average no. of neighbours = 2.0, characteristic path length = 3.2) across eight connected components with a network diameter of seven edges (Table S2). Whereas, the resulting Windmill Islands network consisted of 58 nodes (clustering coefficient = 0.4) and 201 edges (average no. of neighbours = 6.9, characteristic path length = 2.4) across three connected components with a network diameter of six edges (Table S2).

Notable associations within the Vestfold Hills network included positive associations between *Saccharibacteria* (*TM7*), a parasitic bacterium, and *Actinobacteria*. Also noted was the lack of co-occurrent micro-eukaryotic species. *Crenarchaeota* were more strongly embedded within the Windmill Islands network suggesting different life histories or niche preferences between the two regions. Similarly, rare candidate bacterial phyla *Candidatus Eremiobacteraeota* (*WPS*-*2*) and *Candidatus Dormibacteraeota* (*AD3*) only formed strong visible associations in this region. The astounding taxonomic diversity of *Actinobacteria* (Fig. 1 and S2) was reflected in their ability to occupy multiple niches and form the majority of connections to co-existing species, essentially moulding the microbial backbone of these Antarctic desert soils. Overall, microorganisms present within the soil microbial networks tended to co-occur more than expected by chance (*P* &lt; 0.001).

### Correlations between estimated richness and selected environmental predictor variables

Generalised additive models (GAMs) were fitted to test the ability of a range of soil parameters to explain the variation in Chao1 richness of bacteria, eukarya and archaea. A stepwise model selection process (based on the lowest AIC) was used to identify the ‘best model’ and thereby identify the key environmental drivers. These models explained a moderate percentage of variation (45.0–66.8%) in richness for all three microbial communities at the regional scale (Table 1 and Fig. S4, S5,S6). For bacteria, there was a positive relationship between Chao1 richness and copper (Cu), aluminium (Al, Al_2_O_3_) and gravel content (Fig. S4). Micro-eukaryote richness exhibited negative relationships with dry matter fraction (DMF), soil pH, nitrite concentrations (NO_3_) and the amount of mud but displayed a positive relationship with total carbon content (TC) and conductivity (Fig. S5). Archaeal richness had positive relationships with conductivity and total nitrogen content (TN) but displayed a negative relationship with calcium (CECCa) (Fig. S6). Both bacteria and archaea showed a positive relationship with phosphorous (TP, P) and sodium (CECNa) but had a negative relationship with titanium dioxide (TiO_2_). Only micro-eukaryotes demonstrated a significant (*P* &lt; 0.05) difference between the two regions.

**Table 1:** Summary of best model selection after the removal of co-variates with region as a random effect

Response	Bacterial Chao1	Eukaryotic Chao1	Archaeal Chao1
Predictor 1	Total phosphorous	Dry matter fraction	Conductivity
Predictor 2	Phosphorous	Conductivity	Total nitrogen
Predictor 3	Copper	pH	Phosphorous
Predictor 4	Aluminium	Total Carbon	Calcium Cation
Predictor 5	Sodium cation	NO_2_	Sodium Cation
Predictor 6	Gravel	Mud	Mud
Predictor 7	TiO_2_	Region	TiO_2_
Predictor 8	Al_2_O_3_		
Distribution	Negative binomial	Gaussian	Gaussian
*R*^2^	0.601	0.34	0.611
Deviance Explained	64.90%	45%	66.80%

### Niche or neutral?

**Fig. 4:**
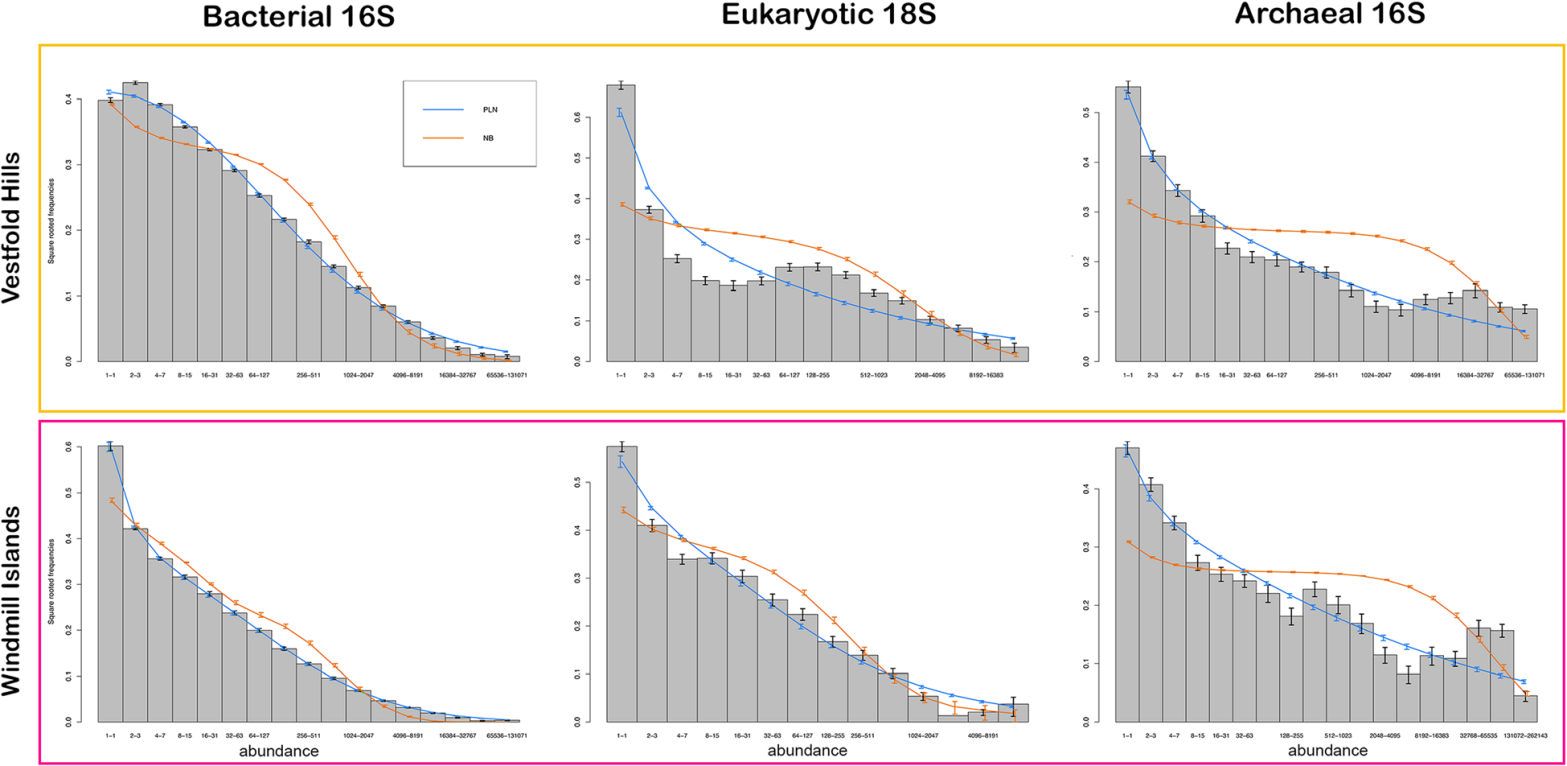
Fitted species abundance distribution (SAD) curves of polar soil microbial communities between the Vestfold Hills and Windmill Islands. The bars represent the mean proportion of species at each site in different octave classes of abundance. The blue and orange lines show the mean of fitted values from region-by-region fits of the Poisson-lognormal (PLN) and negative binomial (NB) distributions to the data, respectively. A PLN-fit best explains the overall structure of these communities, particularly for bacterial communities at the Windmill Islands. Eukaryotic and archaeal communities demonstrate slightly weaker PLN-fits and multimodal distributions across both regions, suggesting the emergence of neutrality

Overall, species abundances were better approximated by Poisson-lognormal (PLN) than negative binomial (NB) distributions (_w_PLN&gt;_w_NB), likely attributable to these Antarctic communities being substantially more heterogenous than expected (Fig. 4, Table 2). As is the norm in ecological communities, all distributions were characterised by highly right-skewed patterns, emphasising the disparity between rare and common species. Bacterial communities lacked an internal mode and demonstrated a better PLN-fit (Table 2), particularly at the Windmill Islands (_w_PLN = 1.000, _w_NB = &lt; 0.001). By contrast, eukaryotic and archaeal communities demonstrated multimodal distributions accompanied by relatively weaker PLN-fits, particularly for eukaryotic communities at the Vestfold Hills (_w_PLN = &lt; 0.001, _w_NB = 1.000). These trends remained consistent at the local scale (Fig. S7, Table S4).

**Table 2:** Akaike weights (AIC) calculated from regional-scale PLN- and NB-fitted SADs (where a weighted value closer to 1 indicates stronger evidence of one model over the other)

	Dataset	wPLN	wNB
Bacteria	Windmill Islands	1.000	&lt; 0.001
	Vestfold Hills	1.000	&lt; 0.001
Eukarya	Windmill Islands	0.917	0.083
	Vestfold Hills	&lt; 0.001	1.00
Archaea	Windmill Islands	1.000	&lt; 0.001
	Vestfold Hills	0.960	0.0405

## Discussion

Akin to other arid soil environments around the globe (Cowan et al. 2014), this extensive survey of the east Antarctic soil microbiome reveals that whilst bacterial diversity is rich, both micro-eukaryotic and archaeal phylogenies were comparatively low (Figs. 1 and 3). Overall, bacterial communities were dominated by the metabolically and physiologically diverse *Actinobacteria* phylum. Their ubiquity throughout terrestrial and aquatic ecosystems, including extreme environments like Antarctica, is a direct reflection of their genomic heterogeneity and broad functional capacities [6, 31]. However, regional disparity amongst taxa between the Vestfold Hills and Windmill Islands was observed. The Vestfold Hills, a region comprising of low-lying hilly country indented by lakes, contained a higher prevalence of bacterial members belonging to the *Bacteroidetes* phylum. This is likely due to its comparatively higher salinity levels than the Windmill Islands (Table S3), manifesting as visible salt crystal encrustations on the soil surface. In contrast, rare bacterial candidate phyla *Eremiobacteraeota* (WPS-2) and *Dormibacteraeota* (AD3), previously implicated in a novel mode of primary production using atmospheric energy sources [6], were notably lower in abundance at the Vestfold Hills. Possibly due to the relatively higher proportion of micro-eukaryotic taxa capable of photosynthesis in this region, namely phylum *Ochrophyta* and *Ciliophora* (Fig. 1 and S2). In contrast, archaeal communities, being mainly distributed within the *Crenarchaeota* or *Thaumarchaeota* phylum, were ubiquitous across both regions. Members in this phylum, more specifically *Nitrososphaera*, are known for their ammonia oxidising capabilities [32], thereby further highlighting the ecological importance of the vastly understudied polar soil archaea. Although we have only begun to shed light on the hidden complexities of the Antarctic soil microbiome, it is an important step towards achieving an integrated understanding of the basic ecological mechanisms governing these assemblages within such a severely limiting environment.

Strong niche partitioning appear to be driving the establishment and maintenance of contemporary microbiomes of the arid-to-hyperarid east Antarctic soils analysed here (Fig. 4; Table 2). This was particularly evident for bacterial communities at the Windmill Islands, where environmental gradients, such as soil pH and DMF, were generally more pronounced between sites (Fig. S3; Table S3). Whereas, soil parameters between sites at the Vestfold Hills were more similar to one another, with the exception of The Ridge (TR) (Fig. S3). These regional differences are also reflected in their phylogenetic composition, abundance and richness of microbial taxa (Figs. 1 and 3). Reduced niche overlap likely promotes greater biodiversity and long-term species co-existence through the efficient exploitation of resources under the adverse conditions [26, 33]. This may attribute to our observations of high bacterial diversity whilst both eukaryotic and archaeal diversities were relatively low (Figs. 1 and 3). Communities also demonstrated mixed responses to soil environmental predictors such as fertility and grain-related factors as well as metal oxide concentrations, which is likely a reflection of varied life history strategies (Table 1). Most notably, regional effects were only significant in explaining variation in richness for micro-eukarya, suggesting that other influences such as dispersal limitation may come into play for micro-eukaryotic communities between the Vestfold Hills and Windmill Islands [34, 35, 36].

In an era of progressively rapid natural and anthropogenic change, communities demonstrating strong niche-driven responses may have increased susceptibility to disturbance events such as large-scale colonisation, like those observed by *Rhizocarpon* lichens and invasive grass *Poa annua* across the Antarctic Peninsula (Chown et al. 2012 [2];; Supp and Ernest 2015). Inevitably, this will alter contemporary ecosystem dynamics and potentially result in the loss of novel polar taxa and associated traits due to the reduced functional insurance of strongly niche-shaped communities [20, 27, 37, 38]. For example, *Candidatus Eremiobacteraeota* (*WPS-2*) and *Candidatus Dormibacteraeota* (*AD3*) who are comprised of members genetically capable of atmospheric chemosynthesis, a novel process proposed to be contributing to primary production in these nutrient poor desert soils [6].

Neutral processes, however, play larger-than-expected roles within the eukaryotic and archaeal soil communities analysed, particularly throughout the Vestfold Hills (Fig. 4 and S5; Table 2 and S5). Weaker PLN-fits and apparent SAD multimodality suggest the emergence of neutrality for functionally similar groups [39–42] like *Nitrososphaera* (Fig. S2), a genus of chemotrophic ammonia oxidisers, likely involved in nitrogen cycling within these nutrient-limited Antarctic soils [43]. Interestingly, draft genomes of *Thaumarchaeota* recovered from Robinson Ridge (RR) soils reported the presence of ammonia monooxygenase [6], the first enzyme in the pathway for nitrification [44], further implicating the functional relevance of archaea in polar soils (Fig. 3). Moreover, members forming metabolic alliances with or competing against co-occurring bacterial taxa, such as *Crenarchaeota* at the Windmill Islands and micro-eukarya in general at the Vestfold Hills (Fig. 3), are likely critical to the formation of functional microbiomes within these harsh environments ([45]; Bahram et al. 2018 [20];). Unless competition is a major driving force within the relatively species poor eukaryotic and archaeal communities, their emerging neutral status may also promote greater resilience against perturbations due to their ephemeral natures, which is perhaps a cyclic response to seasonal resource availability, such as increased water and nutrient bioavailability during the austral summer [26, 46].

Although there is no current consensus on what drives SAD shape variation [30], a number of studies argue that multimodality occurs quite frequently in nature, and as such it is indeed a characteristic of ecological communities [39, 40, 42]. Emergent neutrality is one hypothesis put forth to explain multimodal SADs, where transient self-organised patterns of functionally similar species coexist within an ecological niche [41, 42]. Other studies claim that multimodality may arise from sampling artefacts [47]. We acknowledge that potential biases may be introduced through amplicon sequencing due to limitations in primer design but multimodality is rarely reported and its implications poorly understood, thus these findings warrant further consideration [39].

## Conclusions

Information on biodiversity, assemblage patterns, environmental drivers and non-random co-occurrences are extremely valuable for Antarctic soil ecosystems, particularly the Vestfold Hills, where the basic diversity, ecology and life history strategies of resident microbiota is limited [48, 49]. These findings provide a new understanding of the basic ecological concepts underlying Antarctic species abundance and distribution. Regional disparities between soil communities at the Vestfold Hills and Windmill Islands further support the notion that microbial biogeography exists. Thus, stressing the importance of conserving these unique ecologies in the face of a warming Antarctica. Furthermore, spatial and temporal shifts in the community SAD patterns documented here can potentially be used to infer responses to environmental disturbance, before any local extinctions can occur at the micro-biodiversity scale.

## Methods

### Study area, soil sampling and physiochemical analysis

**Fig. 5:**
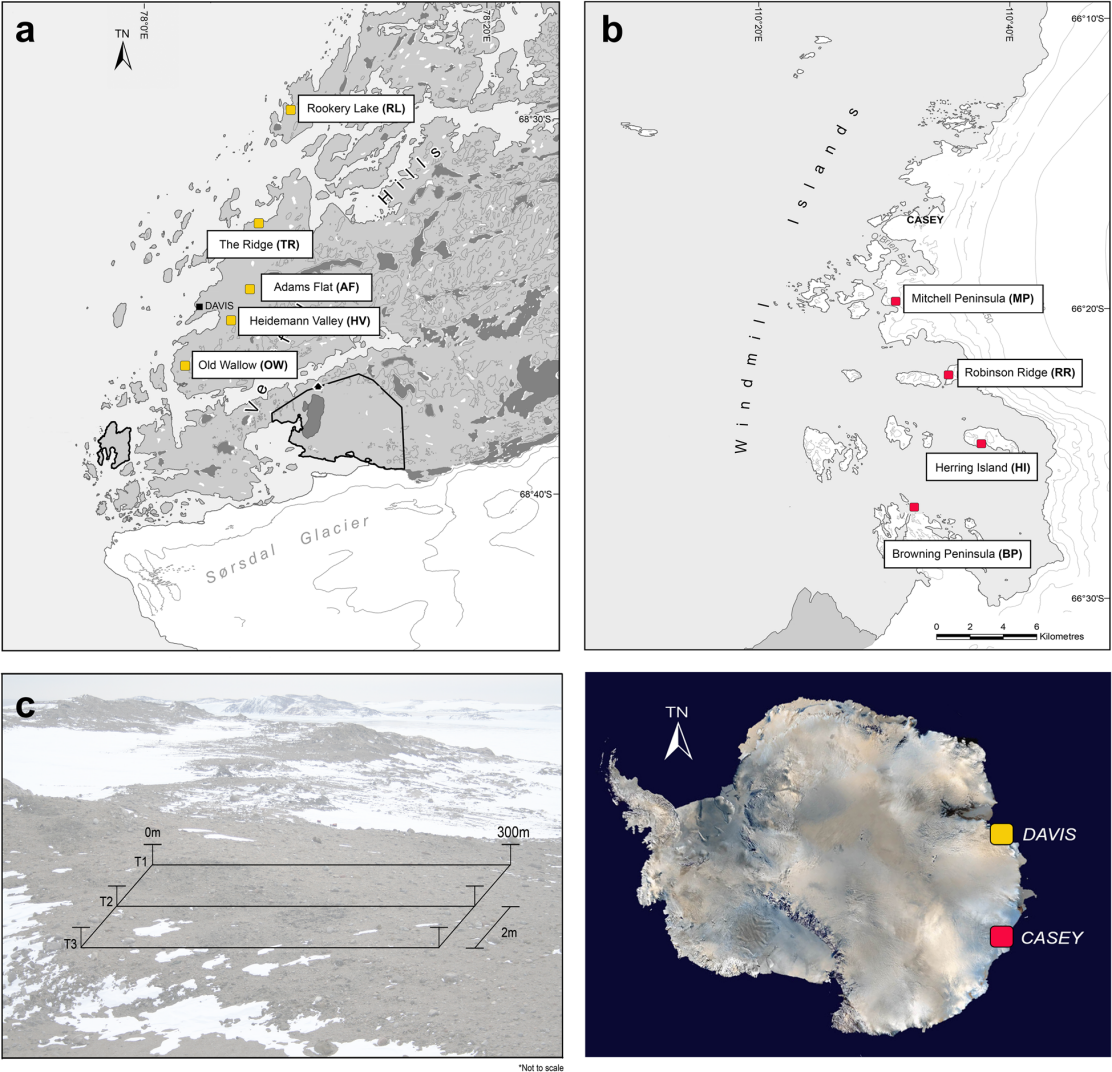
Map of the nine study areas across the **a** Vestfold Hills (AAD map catalogue No. 14, 499) and **b** Windmill Islands (No. 14, 179) region of Eastern Antarctica, showing approximate sampling locations and **c** geospatial transect design. At each site, soil samples (*n* = 93) were taken at the following distance points along each transect: 0, 0.1, 0.2, 0.5, 1, 2, 5, 10, 20, 50, 100, 100.1, 100.2, 100.5, 101, 102, 105, 110, 120, 150, 200, 200.1, 200.2, 200.5, 201, 202, 205, 210, 220, 250 and 300m. Where underlined distance points refer to a subsample (*n* = 18) submitted for amplicon sequencing of eukaryotic (18S rRNA) and archaeal (16S rRNA) soil communities

Sampling was performed by expeditioners via the Australian Antarctic Program (AAP) across nine polar desert sites spanning two ice-free regions (the Vestfold Hills and Windmill Islands). All nine sites were within the vicinity of Casey (66° 17′ S, 110° 45′ E) and Davis (68° 35′ S, 77° 58′ E) research stations in Eastern Antarctica (Fig. 4). Five sites were chosen from the Vestfold Hills: Adams Flat (AF: 68° 33′ S, 78° 1′ E); Old Wallow (OW: 68° 36′ S, 77° 58′ E); Rookery Lake (RL: 68° 36′ S, 77° 57′ E); Heidemann Valley (HV: 68° 35′ S, 78° 0′ E); and The Ridge (TR: 68° 54′ S, 78° 07′ E). Four sites were chosen from the Windmill Islands: Mitchell Peninsula (MP: 66° 31′ S, 110° 59′ E); Browning Peninsula (BP: 66° 27′ S, 110° 32′ E); Robinson Ridge (RR: 66° 22′ S, 110° 35′ E); and Herring Island (HI: 66° 24′ S, 110° 39′ E). At each site, soil samples (*n* = 93) from the top 10 cm were taken along three parallel transects following a geospatially explicit design [7]. All soils (*n* = 837) included in this study were previously submitted for extensive chemical and physical attributes (Table S2) [7, 50] (Fig. 5).

### DNA extraction and Illumina amplicon sequencing

DNA was extracted in triplicate from soil samples using the FASTDNA™ SPIN Kit for Soil (MP Biomedicals, Santa Ana, CA, USA) and quantified using the Qubit™ 4 Fluorometer (ThermoFisher Scientific, NSW, Australia) as described in van Dorst et al. 2014. Diluted DNA (1:10 using nuclease-free water) was submitted to the Ramaciotti Centre for Genomics (UNSW, Sydney, Australia) for amplicon paired-end sequencing on the Illumina MiSeq platform (Illumina, California, USA) with negative and positive (mock) controls in accordance to protocols from Bioplatforms Australia (BPA) [50]. All 93 samples from each site were submitted to sequencing for bacteria (*n* = 837) targeting the 16s rRNA gene using the 27F/519R [51] primer set. As described in Siciliano et al. [7], a non-random subsample (*n* = 18) at each site was selected for the sequencing of micro-eukarya (*n* = 162) and archaea (*n* = 144) targeting the 18S and 16S rRNA genes using the 1391f/EukBr (Amaral-Zettler et al. 2009) and A2F/519R [52] primer sets, respectively. These include distance points at 0, 2, 100, 102, 200 and 202 m along each of the three parallel transects at each site.

### Open OTU picking, assignment and classification

We followed the UPARSE OTU algorithm [53] endorsed by BPA through directly employing USEARCH 32-bit v10.0.240 [54] and VSEARCH 64-bit v2.8.0 [55]. Sequences were quality filtered, trimmed and clustered de novo to pick OTUs at 97% identity. Reads were then assigned to separate sample-by-OTU matrices for each amplicon (Table S1). OTUs were taxonomically classified against the SILVA v3.2.1 SSU rRNA database [56]. Where applicable, new OTU matrices were merged with existing ones using the QIIME 2 (https://qiime2.org) feature-table merge option. These were rarefied using the qiime feature-table rarefy function to generate random subsamples (bacterial 16S rRNA gene = 700k reads, micro-eukaryotic 18S rRNA gene = 23k reads, archaeal 16S rRNA gene = 850k reads).

### Multivariate and statistical analyses in R

All multivariate and statistical analyses were carried out in the R environment (R Core Team 2018) using the subsampled datasets for bacteria, micro-eukarya and archaea. Subsampled rarefaction curves (*q* = 0) were generated using the iNEXT package. Non-metric multidimensional scaling (NMDS) ordinations (distance = ‘Euclidean’ for environmental data and distance = ‘Bray-Curtis’ for OTU abundance data) and Chao1 richness estimates were calculated in vegan v2.5-3 [57]. Unless specified otherwise, all plots were visualised using a combination of ggplot2 v3.1.0 [58] and ggpubr v0.2 [59].

### Removal of environmental co-variates and model selection

To try and better understand the relationship between a range of environmental co-variates, we first constructed a Pearson correlation matrix to identify co-correlated variables (*R* &gt; 0.6), and one of each correlated pair was removed from the list of potential predictors. Models were then fitted with all predictors (saturated models) using each of the Chao1 richness variables (bacteria, micro-eukarya and archaea) as the response variable. Starting with this saturated model, the best model (i.e. the most parsimonious—as indicated by the lowest AIC) was then identified using the stepAIC function in the MASS v7.3-51.4 package [60] in R. We also included region (i.e. Windmill Island or Vestfold Hills) as a random effect in the model selection process, to help in understanding the regional effects in explaining variation in richness. We fitted both generalised linear models (GLMs) and generalised additive models (GAMs) with smooth terms as either Gaussian or NB distributions. In addition to AIC, model diagnostic plots (to test normality and heteroscedasticity of variance) were used to help inform final model selection, especially with regard to the distribution used.

### Domain-level co-occurrence OTU network from abundance data

OTUs representing &gt; 0.001% of the total relative abundance of the bacterial, eukaryotic and archaeal communities within each region were combined for network analyses. Correlations between the relative abundance of each OTU pair across samples were calculated using the maximal information coefficient (*MIC*) in the MINE software package [61]. After correction for multiple testing, statistically significant (*P* &lt; 0.001) co-occurrence relationships between pairs of OTUs were uploaded into the CYTOSCAPE software [62] to generate network diagrams, displaying only very strong associations (*MIC* &gt; 0.8). Statistical inferences of network topology were calculated using the Network Analyser algorithm (treatment = ‘undirected’) in CYTOSCAPE (Table S2).

### PLN- and NB-fitted species abundance distribution curves

As described in [63], PLN and NB models representing niche and neutral distributions, respectively, were fitted to our empirical data using maximum likelihood methods. All available samples for bacteria, micro-eukarya and archaea were included in this analysis. Pooled species abundances were fitted on both regional and local scales then displayed on a logarithmic scale. Akaike weights (_w_PLN and _w_NB) were calculated for PLN- and NB-fits on each dataset to provide a measure of the relative goodness for fit [64].

## Supplementary information

**Additional file 1: Figure S1.** Rarefaction curves of subsampled bacterial, eukaryotic and archaeal communities between sites. In all cases, data was approaching asymptote indicating that sufficient sampling depth was achieved. A particularly rich number of bacterial, eukaryotic and archaeal species were observed at MP (Mitchell Peninsula), TR (The Ridge) and RR (Robinson Ridge), respectively. **Figure S2.** Top 15 most genus of bacterial, eukaryotic and archaeal communities between sites. As taxonomic levels decrease, the number of unclassified taxa increase substantially. Interestingly, archaeal communities were dominated by *Nitrososphaera*, a genus of ammonia oxidising archaea possibly implicated in nitrogen cycling within these nutrient starved soils. **Figure S3.** NMDS plots of microbial OTU communities and environmental soil parameters. In all cases, soil samples clustered according to site and broadly by geographic region. Although TR (The Ridge) is more environmentally similar to the Windmill Island sites, it’s soil bacterial and eukaryotic communities cluster more strongly with the Vestfold Hills. **Figure S4.** GAM model output of negative binomial distributions of best environmental predictor variables against estimated bacterial Chao1 richness based on AIC, where ‘*’ indicates a significant (*P*&lt;0.05) correlation. A positive relationship is generally observed between bacterial richness and copper (CU), phosphorous (TP, P), aluminium (AL, AL_2_O_3_), sodium ion concentrations (CECNA) and the amount of gravel (GRVL) but displayed a negative relationship with titanium dioxide (TIO_2_). **Figure S5.** GAM model output of gaussian distributions of best environmental predictor variables against estimated eukaryotic Chao1 richness based on AIC, where ‘*’ indicates a significant (*P*&lt;0.05) correlation. A negative relationship is generally observed between eukaryotic richness and dry matter fraction (DMF), soil pH, nitrite concentrations (NO_2_) and mud content but displayed a positive relationship with total carbon (TC) and conductivity (COND). A significant correlation is observed against random regional effects. **Figure S6.** GAM model output of gaussian distributions of best environmental predictor variables against estimated archaeal Chao1 richness based on AIC, where ‘*’ indicates a significant (*P*&lt;0.05) correlation. Archaeal richness displayed positive relationships with conductivity (COND), total nitrogen (TN), phosphorous (TP, P) and sodium ion concentrations (CECNA), whilst a negative relationship was observed against titanium dioxide (TIO_2_). **Figure S7.** Local scale PLN- (blue) and NB-fitted (orange) SADs of the nine sites studied. These trends remain consistent with those observed for the regional fitted SADs, where bacterial communities display strong niche-driven signatures across all sites whilst eukaryotic and archaeal communities demonstrated weaker PLN-fits and multimodality. **Table S1.** Summary of amplicon sequencing output and OTU pipeline analysis. Table S2 CYTOSCAPE network topology analysis between regions at the domain-level. **Table S3.** Environmental soil parameters averaged between sites. **Table S4.** Akaike weights calculated from local-scale PLN- and NB-fitted SADs. Where NA indicates that the fitting procedure did not converge, which is usual for small datasets.

## Data Availability

The datasets generated and analysed during the current study are available through the Australian Antarctic Data (AAD) Centre, [10.4225/15/526F42ADA05B1] and the AusMicrobiome repository, [https://data.bioplatforms.com/organization/about/australian-microbiome]. Please contact author for additional data requests.
