# Supplementary material for: Lifting the veil on arid-to-hyperarid Antarctic soil microbiomes: a tale of two oases
Source: Microbiome. 2020 Mar 16;8:37. doi: 10.1186/s40168-020-00809-w (PMC7076931; doi:10.1186/s40168-020-00809-w)
Supplement: Supplementary file 1 — Additional file 1: Figure S1. Rarefaction curves of subsampled bacterial, eukaryotic and archaeal communities between sites. In all cases, data was approaching asymptote indicating that sufficient sampling depth was achieved. A particularly rich number of bacterial, eukaryotic and archaeal species were observed at MP (Mitchell Peninsula), TR (The Ridge) and RR (Robinson Ridge), respectively. Figure S2. Top 15 most genus of bacterial, eukaryotic and archaeal communities between sites. As taxonomic levels decrease, the number of unclassified taxa increase substantially. Interestingly, archaeal communities were dominated by Nitrososphaera, a genus of ammonia oxidising archaea possibly implicated in nitrogen cycling within these nutrient starved soils. Figure S3. NMDS plots of microbial OTU communities and environmental soil parameters. In all cases, soil samples clustered according to site and broadly by geographic region. Although TR (The Ridge) is more environmentally similar to the Windmill Island sites, it’s soil bacterial and eukaryotic communities cluster more strongly with the Vestfold Hills. Figure S4. GAM model output of negative binomial distributions of best environmental predictor variables against estimated bacterial Chao1 richness based on AIC, where ‘*’ indicates a significant (P<0.05) correlation. A positive relationship is generally observed between bacterial richness and copper (CU), phosphorous (TP, P), aluminium (AL, AL2O3), sodium ion concentrations (CECNA) and the amount of gravel (GRVL) but displayed a negative relationship with titanium dioxide (TIO2). Figure S5. GAM model output of gaussian distributions of best environmental predictor variables against estimated eukaryotic Chao1 richness based on AIC, where ‘*’ indicates a significant (P<0.05) correlation. A negative relationship is generally observed between eukaryotic richness and dry matter fraction (DMF), soil pH, nitrite concentrations (NO2) and mud content but displayed a positive [file 40168_2020_809_MOESM1_ESM.docx]

Supplementary Information for

**Lifting the veil on arid-to-hyperarid Antarctic soil microbiomes: A tale of two oases**

**Eden Zhang, Loïc M. Thibaut, Aleks Terauds, Mark M. Tanaka, Josie van Dorst, Sin Y. Wong, Sally Crane, Belinda C. Ferrari^*^**

^*^To whom correspondence should be addressed. E-mail: b.ferrari@unsw.edu.au

**This file contains**

Figures S1 to S7

Tables S1 to S4

**Supplementary figures and legends**

**
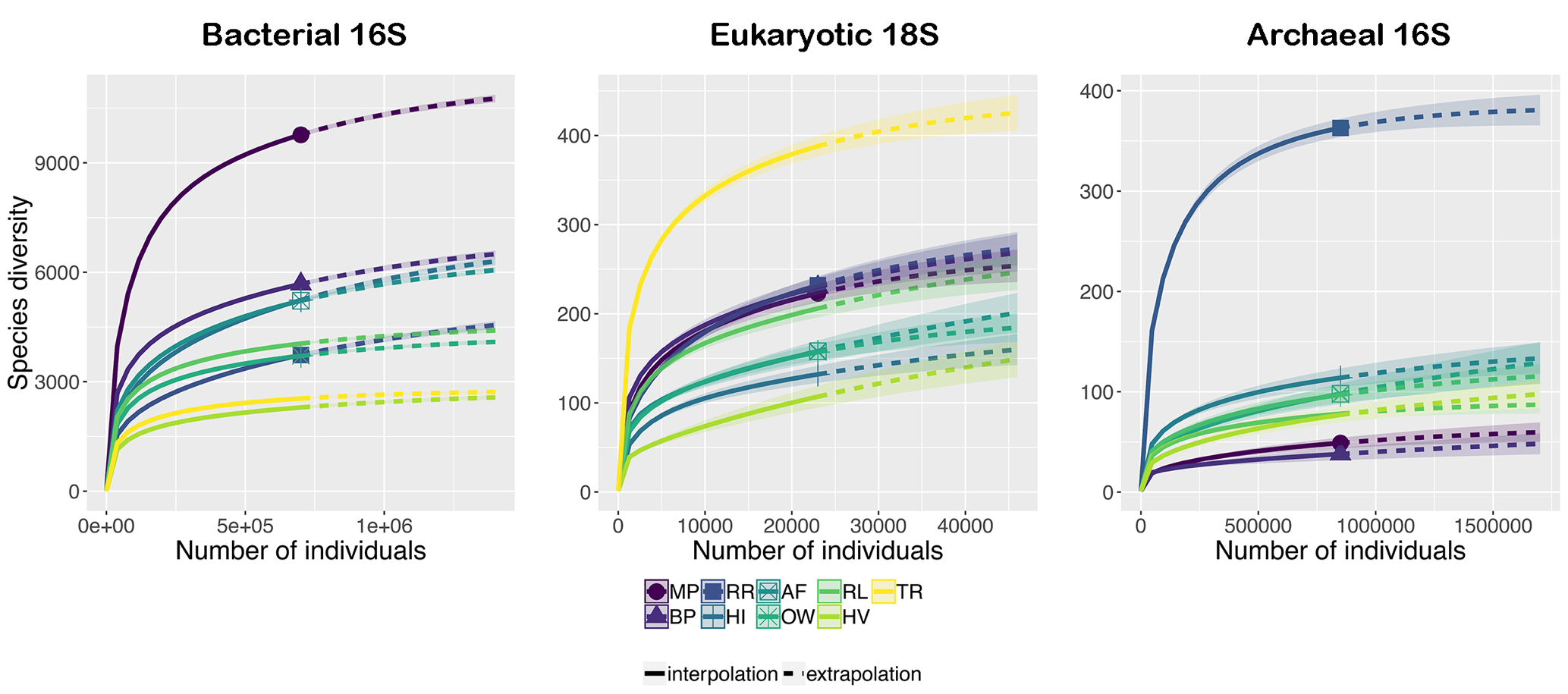
**

**Figure S1** **Rarefaction curves of subsampled bacterial, eukaryotic and archaeal communities between sites.** In all cases, data was approaching asymptote indicating that sufficient sampling depth was achieved. A particularly rich number of bacterial, eukaryotic and archaeal species were observed at MP (Mitchell Peninsula), TR (The Ridge) and RR (Robinson Ridge), respectively.

**
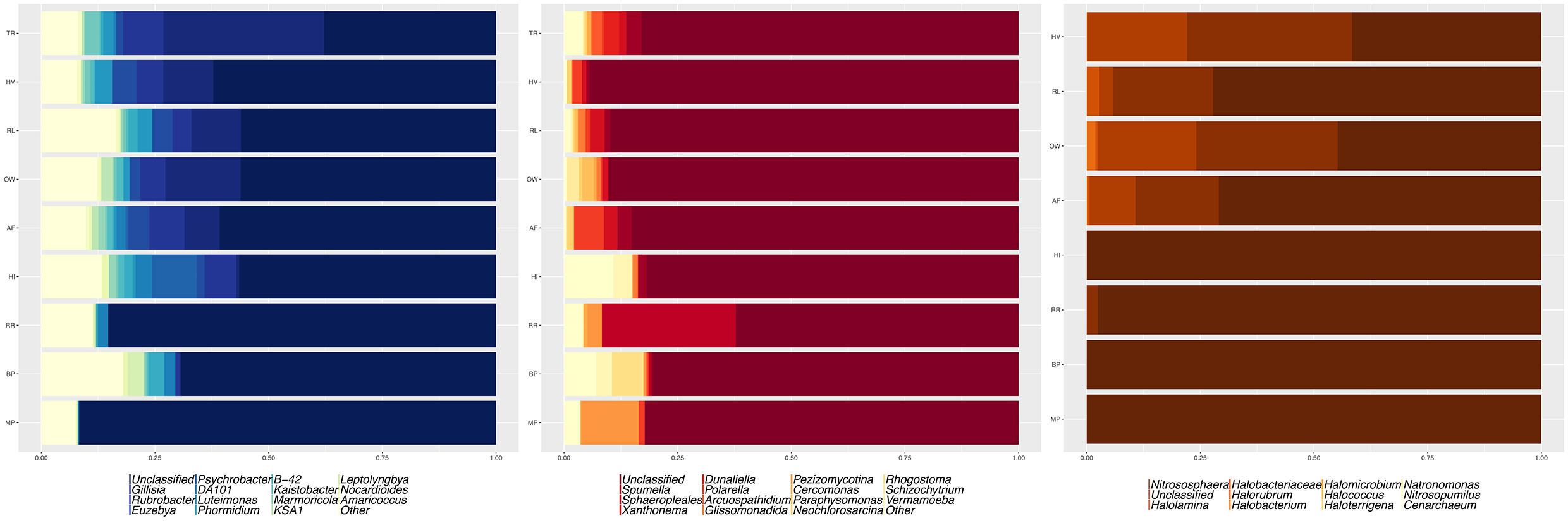
**

**Figure S2** **Top 15 most genus of bacterial, eukaryotic and archaeal communities between sites.** As taxonomic levels decrease, the number of unclassified taxa increase substantially. Interestingly, archaeal communities were dominated by *Nitrososphaera*, a genus of ammonia oxidising archaea possibly implicated in nitrogen cycling within these nutrient starved soils.

**
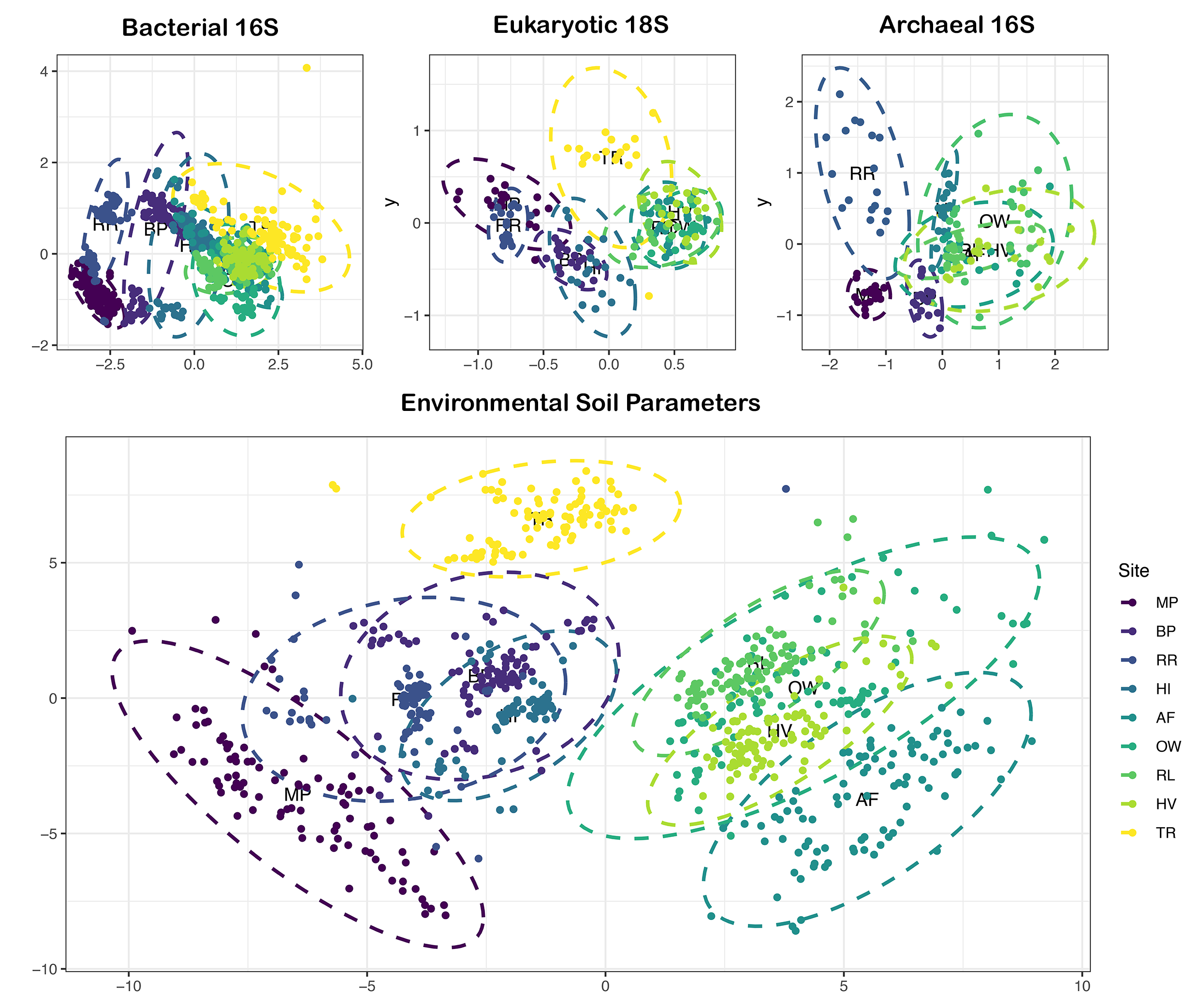
**

**Figure S3** **NMDS plots of microbial OTU communities and environmental soil parameters.** In all cases, soil samples clustered according to site and broadly by geographic region. Although TR (The Ridge) is more environmentally similar to the Windmill Island sites, it’s soil bacterial and eukaryotic communities cluster more strongly with the Vestfold Hills.


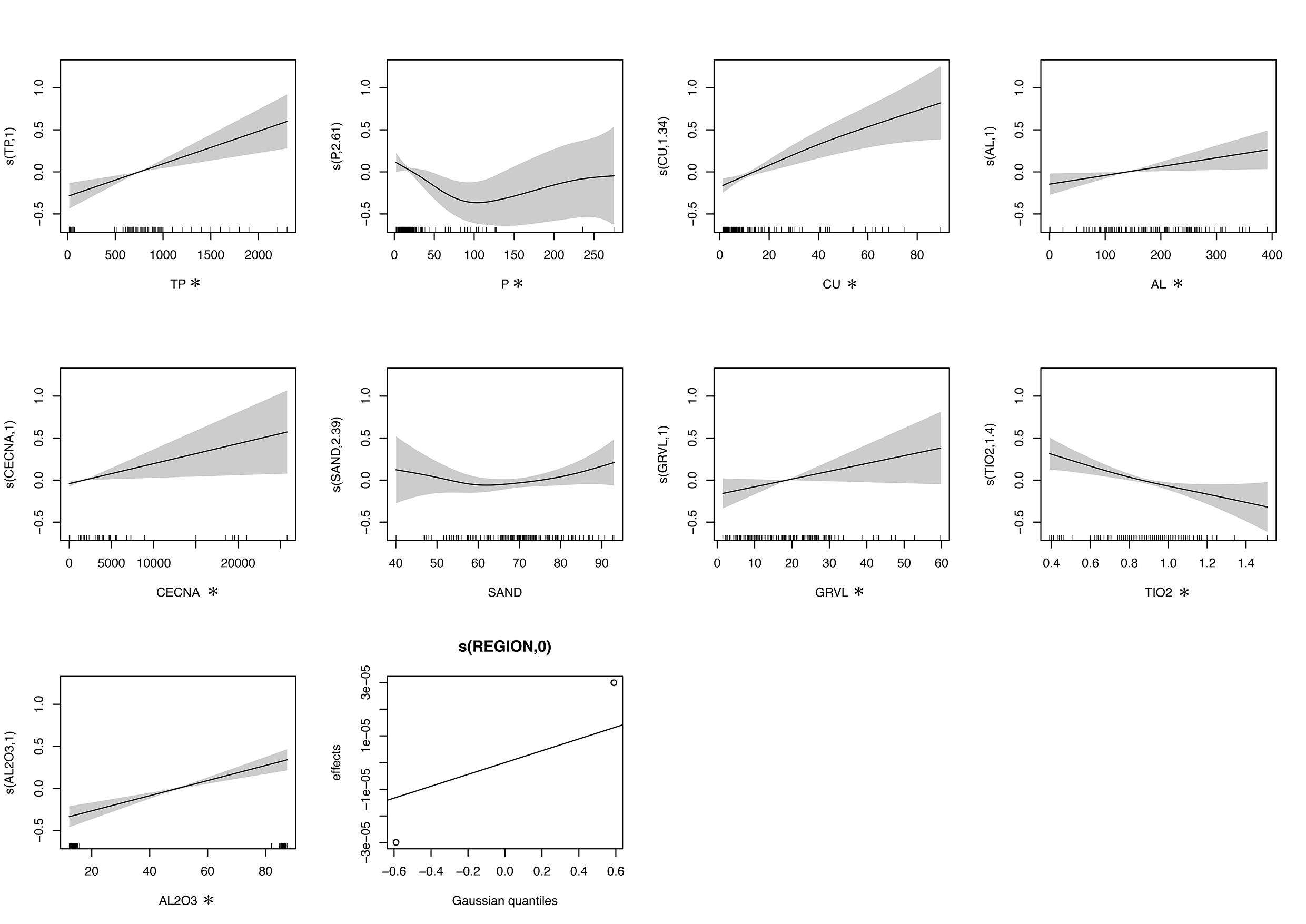


**Figure S4** **GAM model output of negative binomial distributions of best environmental predictor variables against estimated bacterial Chao1 richness based on AIC, where ‘*’ indicates a significant (*P*<0.05) correlation.** A positive relationship is generally observed between bacterial richness and copper (CU), phosphorous (TP, P), aluminium (AL, AL_2_O_3_), sodium ion concentrations (CECNA) and the amount of gravel (GRVL) but displayed a negative relationship with titanium dioxide (TIO_2_).


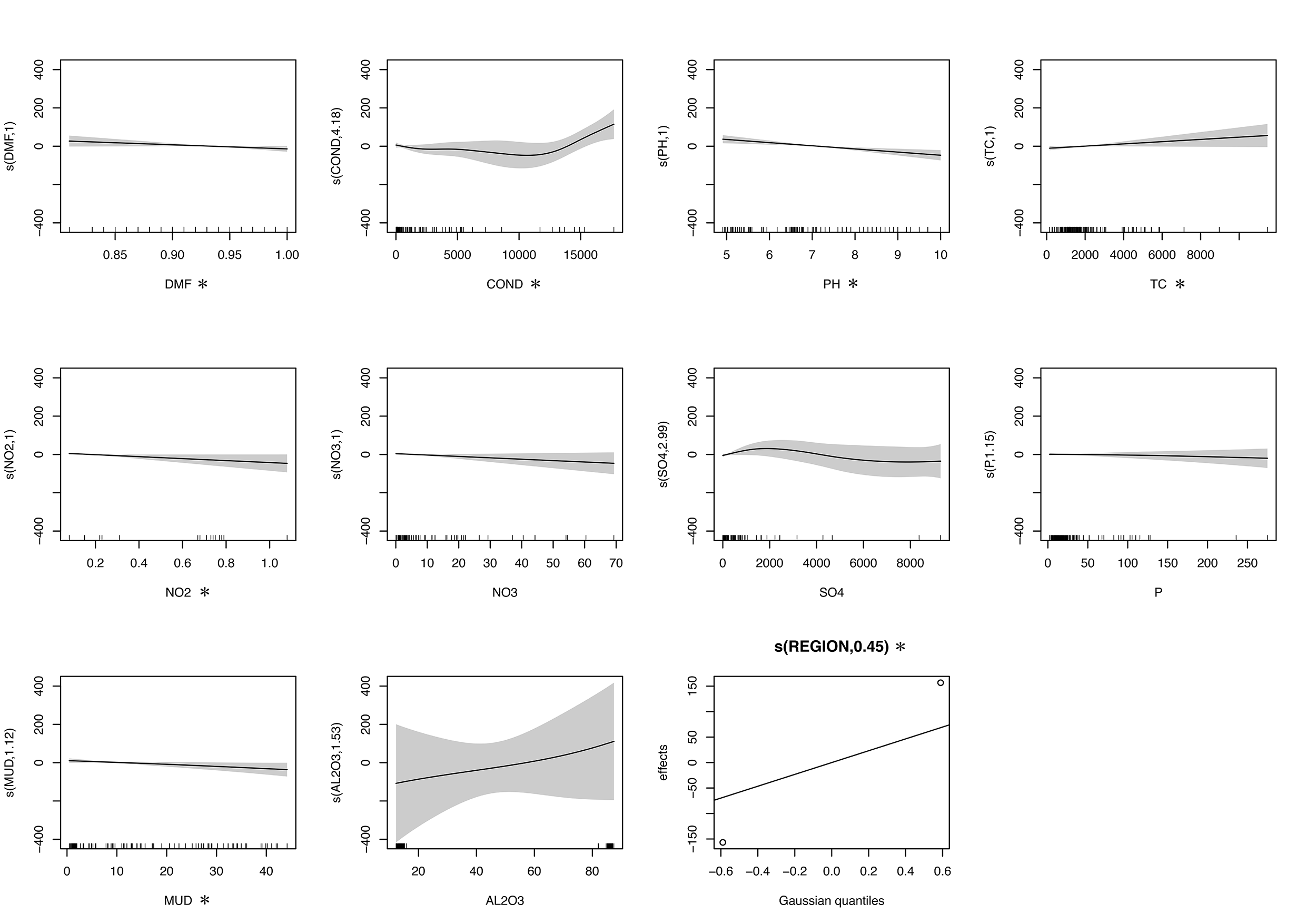


**Figure S5** **GAM model output of gaussian distributions of best environmental predictor variables against estimated eukaryotic Chao1 richness based on AIC, where ‘*’ indicates a significant (*P*<0.05) correlation.** A negative relationship is generally observed between eukaryotic richness and dry matter fraction (DMF), soil pH, nitrite concentrations (NO_2_) and mud content but displayed a positive relationship with total carbon (TC) and conductivity (COND). A significant correlation is observed against random regional effects.


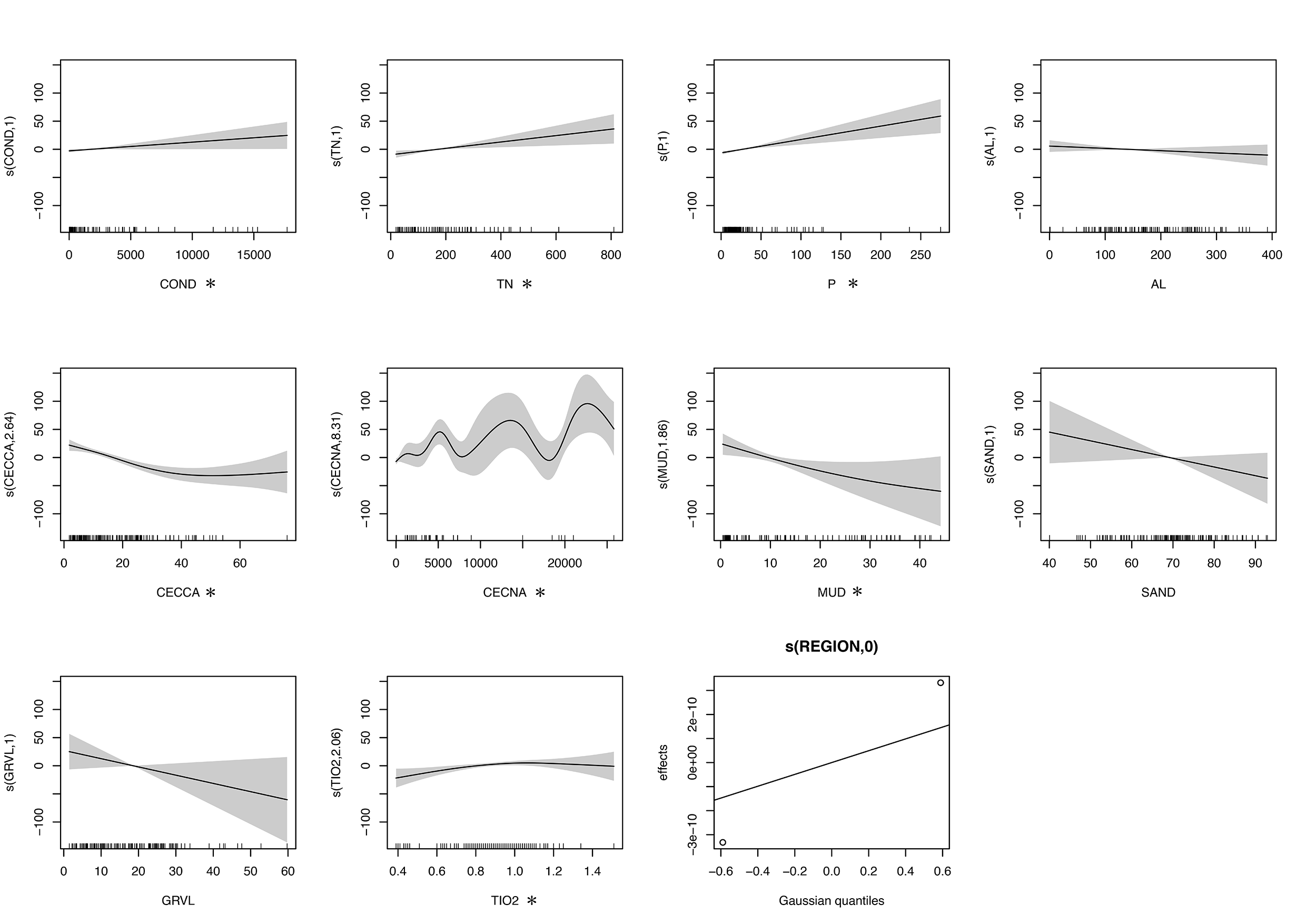


**Figure S6** **GAM model output of gaussian distributions of best environmental predictor variables against estimated archaeal Chao1 richness based on AIC, where ‘*’ indicates a significant (*P*<0.05) correlation.** Archaeal richness displayed positive relationships with conductivity (COND), total nitrogen (TN), phosphorous (TP, P) and sodium ion concentrations (CECNA), whilst a negative relationship was observed against titanium dioxide (TIO_2_).

**
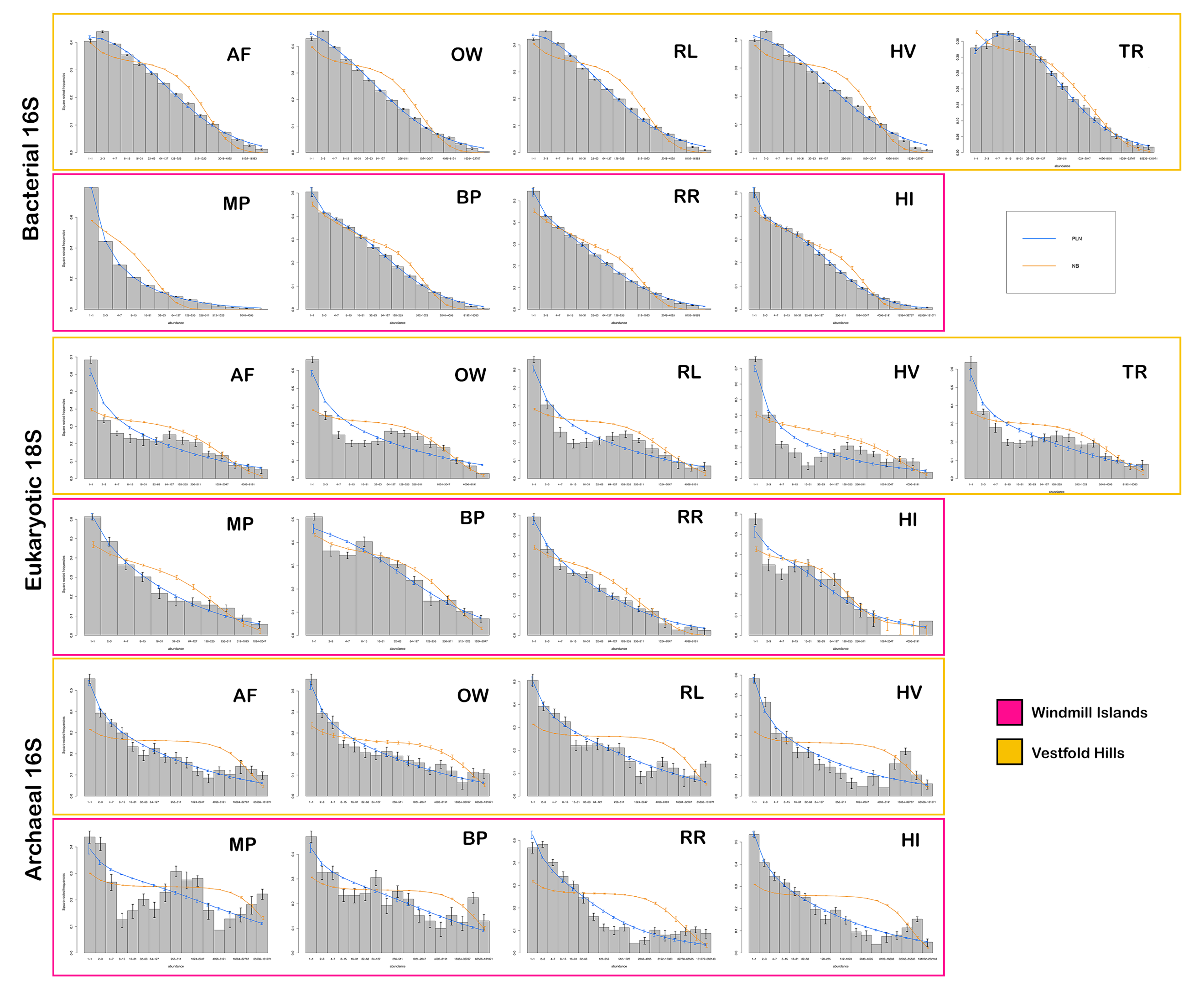
**

**Figure S7 Local scale PLN- (blue) and NB-fitted (orange) SADs of the nine sites studied.** These trends remain consistent with those observed for the regional fitted SADs, where bacterial communities display strong niche-driven signatures across all sites whilst eukaryotic and archaeal communities demonstrated weaker PLN-fits and multimodality.

**Supplementary tables**

**Table S1** **Summary of amplicon sequencing output and OTU pipeline analysis.**

|  | **Per Site** | **Total** | **Reads after Filtering** | **OTUs** | **Singletons** |
| --- | --- | --- | --- | --- | --- |
| **Bacterial 16S** | 93 | 837 | 60, 495, 244 | 36, 251 | 9, 952 |
| **Eukaryotic 18S** | 18 | 162 | 1, 299, 519 | 1, 511 | 329 |
| **Archaeal 16S** | 18 | 144 | 13, 373, 072 | 589 | 72 |

**Table S2** **CYTOSCAPE network topology analysis between regions at the domain-level.**

|  | **Vestfold Hills** | **Windmill Islands** |
| --- | --- | --- |
| **No. of Nodes** | 43 | 58 |
| **No. of Edges** | 44 | 201 |
| **Network Density** | 0.049 | 0.122 |
| **Network Heterogeneity** | 0.567 | 0.873 |
| **Clustering coefficient** | 0.214 | 0.448 |
| **Connected components** | 8 | 3 |
| **Network diameter** | 7 | 6 |
| **Network centralisation** | 0.074 | 0.401 |
| **Shortest paths** | 518 (28%) | 2482 (75%) |
| **Characteristic path length** | 3.247 | 2.377 |
| **Average no. of neighbours** | 2.047 | 6.931 |

**Table S3** **Environmental soil parameters averaged between sites.**

|  | **Parameter** | **MP** | **BP** | **RR** | **HI** | **AF** | **OW** | **RL** | **HV** | **TR** |
| --- | --- | --- | --- | --- | --- | --- | --- | --- | --- | --- |
| **Geographical** | Elevation (m) | 31.76 | 41.24 | 35.67 | 30.92 | 3.01 | 18.9 | 12.13 | 11.68 | 27.91 |
|  | Slope | 9.48 | 2.8 | 6.91 | 6.94 | 0.72 | 1.94 | 3.24 | 1.95 | 5.69 |
|  | Aspect | 217.6 | 172.76 | 119.28 | 130.46 | 137.59 | 188.93 | 165.11 | 143.8 | 201.46 |
|  | DMF | 0.97 | 0.89 | 0.91 | 0.96 | 0.98 | 0.92 | 0.92 | 0.93 | 0.96 |
|  | Conductivity | 49.54 | 36.62 | 23.39 | 163.03 | 2623.94 | 6361.04 | 3061.61 | 1738.61 | 2898.69 |
|  | pH | 5.41 | 6.57 | 5.3 | 6.72 | 7.92 | 8.43 | 7.96 | 8.85 | 8.87 |
| **Nutrients** | TC | 1475.84 | 1938.89 | 2586.56 | 691.67 | 1420.95 | 3942.67 | 1414.59 | 1713.72 | 774.28 |
|  | TN | 46.38 | 184.62 | 235.53 | 152.35 | 132.78 | 303.5 | 168.28 | 140.06 | 81.75 |
|  | TP | 28.48 | 800 | 40.42 | 1069.44 | 981.11 | 707.78 | 1655.56 | 729.44 | 753.26 |
| **Water Extractable Ions** | Cl Water (ppm) | 50.99 | 42.83 | 6.1 | 130.85 | 3899.05 | 4287.37 | 2603.72 | 1783.88 | 1964.78 |
|  | NO_2_ Water (ppm) | 0.08 | 0.08 | 0.13 | 0.08 | 0.76 | 0.31 | 0.54 | 0.7 | 1 |
|  | Br Water (ppm) | 0.08 | 0.11 | 0.14 | 0.19 | 5.34 | 11.3 | 2.42 | 0.95 | NA |
|  | NO_3_ Water (ppm) | 0.35 | 1.75 | 0.67 | 0.87 | 15.1 | 7.06 | 21.94 | 3.95 | 6.09 |
|  | PO_4_ Water (ppm) | 0.63 | 1.17 | 4.65 | 5.06 | 3.86 | 2.07 | 5.04 | 3.71 | 2.06 |
|  | SO_4_ Water (ppm) | 14.15 | 9.57 | 6.94 | 29.76 | 1079.66 | 1489.86 | 375.28 | 634.11 | 1549.56 |
| **Elemental Cation Levels** | P (mg/kg) | 22.43 | 12.17 | 45.11 | 64.56 | 9.12 | 8.96 | 36.09 | 18.54 | NA |
|  | K (mg/kg) | 0.49 | 54.85 | 0.86 | 94.07 | 135.7 | 241.59 | 418.82 | 205.52 | 268.45 |
|  | Ca (mg/kg) | 0.16 | 114.62 | 0.1 | 94.59 | 971.97 | 3283.52 | 842.45 | 1171.69 | 965.67 |
|  | Mg (mg/kg) | 28.17 | 116.92 | 70.31 | 55.13 | 303.92 | 1348.22 | 476.13 | 449.35 | 268.45 |
|  | Zn (mg/kg) | 2.32 | 1.19 | 1.79 | 0.96 | 1.17 | 1.93 | 1.59 | 1.59 | 1.01 |
|  | B (mg/kg) | 136.92 | 0.31 | 377.52 | 0.51 | 4.28 | 4.92 | 5.01 | 5.42 | 9.59 |
|  | S (mg/kg) | 1.28 | 10.02 | 4.82 | 17.86 | 498.17 | 1393.84 | 471.57 | 490.22 | 648.35 |
|  | Cu (mg/kg) | 50.54 | 2.25 | 25.42 | 2.57 | 4.95 | 11.4 | 7.17 | 8.76 | 3.9 |
|  | Fe (mg/kg) | 328.74 | 137.45 | 518.8 | 132.85 | 267.12 | 240.69 | 219.4 | 195.69 | 152.04 |
|  | Mn (mg/kg) | 3.11 | 17.92 | 4.37 | 2.3 | 7.58 | 34.04 | 43.33 | 20.36 | 19.56 |
|  | Na (mg/kg) | 0.59 | 54.22 | 1.07 | 239.31 | 2457.62 | 5124.22 | 3348.4 | 1630.17 | 2148.84 |
|  | Al (mg/kg) | 0.1 | 267.26 | 0.45 | 184.68 | 102.83 | 184.47 | 199.33 | 183.9 | 135.92 |
|  | CECe (meq/100g) | 0.07 | 1.92 | 0.1 | 2.21 | 40.79 | 72.79 | 46.16 | 39.56 | 16.63 |
|  | Ca (meq/100g) | 0.22 | 0.57 | 0.11 | 0.47 | 4.85 | 16.38 | 4.2 | 5.85 | 4.83 |
|  | Mg (meq/100g) | 0.02 | 0.97 | 0.04 | 0.45 | 2.5 | 11.1 | 3.92 | 3.7 | 1.73 |
|  | K (meq/100g) | 16.55 | 0.14 | 37.71 | 0.24 | 0.35 | 0.62 | 1.07 | 0.53 | 0.69 |
|  | Na (meq/100g) | 31.77 | 0.24 | 35.48 | 1.04 | 10.69 | 22.29 | 14.56 | 7.09 | 9.34 |
|  | %CEC Ca | 38.01 | 25.3 | 15.04 | 18.27 | 8.86 | 15.49 | 7.46 | 12.84 | 26.06 |
|  | %CEC Mg | 484.44 | 42.49 | 881.11 | 19.08 | 4.84 | 12.93 | 8.02 | 8.98 | 15.12 |
|  | %CEC K | 608.33 | 6.2 | 1550 | 10.07 | 0.75 | 0.83 | 2.11 | 1.39 | 6.58 |
|  | %CEC Na | 4050.5 | 10.35 | 9672.22 | 39.9 | 20.22 | 23.21 | 25.45 | 15.94 | 49.37 |
| **Particle Size** | Mud % | 1.03 | 1.51 | 1.49 | 1.26 | 7.14 | 24.93 | 23.37 | 25.86 | NA |
|  | Sand % | 65.52 | 71.04 | 69.36 | 69 | 84 | 67.36 | 64.31 | 61.86 | NA |
|  | Gravel % | 32.37 | 23.18 | 25.18 | 28.14 | 8.86 | 7.71 | 12.32 | 12.29 | NA |
|  | Minimum (µm) | 47.09 | 17.35 | 24.94 | 35.97 | 13.94 | 3.04 | 2.96 | 2.22 | NA |
|  | Maximum (µm) | 1248.34 | 1185.89 | 1120.16 | 1193.39 | 1104.52 | 834.81 | 989.32 | 905.01 | NA |
|  | Mean (µm) | 465.04 | 410.21 | 349.29 | 440.94 | 321.54 | 211.32 | 213.49 | 198.67 | NA |
|  | Quartile Deviation | 1.06 | 1.14 | 1.39 | 1.06 | 1.16 | 1.86 | 1.84 | 1.84 | NA |
|  | Sorting Coefficient | 0.48 | 0.45 | 0.39 | 0.48 | 0.45 | 0.28 | 0.29 | 0.28 | NA |
|  | Graphic Skewness | -0.21 | -0.21 | -0.18 | -0.24 | -0.18 | -0.24 | -0.08 | -0.1 | NA |
|  | IGS | -0.25 | -0.31 | -0.22 | -0.29 | -0.24 | -0.28 | -0.14 | -0.16 | NA |
|  | Kurtosis | 0.95 | 1.16 | 0.92 | 0.99 | 1.19 | 0.94 | 0.98 | 0.98 | NA |
| **Oxide Levels** | SiO_2_ % | 33.32 | 31.67 | 35.56 | 36.55 | 62.68 | 60.41 | 57.84 | 61.35 | NA |
|  | TiO_2_ % | 0.61 | 0.93 | 0.98 | 0.93 | 0.8 | 0.84 | 1.07 | 0.86 | 60.15 |
|  | Al_2_O_3_ % | 85.61 | 86.7 | 86.32 | 85.88 | 13.81 | 13.1 | 14.8 | 13.45 | 0.92 |
|  | Fe_2_O_3_ % | 6.6 | 5.89 | 6.27 | 6.89 | 7.85 | 9.45 | 9.73 | 9.42 | 14.24 |
|  | MnO % | 0.25 | 0.11 | 0.13 | 0.14 | 0.12 | 0.14 | 0.12 | 0.14 | 9.19 |
|  | MgO %) | 1.86 | 1.39 | 1.84 | 3.04 | 4.08 | 4.78 | 4.47 | 4.7 | 0.12 |
|  | CaO (%) | 2.39 | 3.3 | 3.88 | 5.3 | 5.33 | 5.24 | 5.32 | 5.25 | 4.45 |
|  | Na_2_O (%) | 2.78 | 2.81 | 2.84 | 2.95 | 3.56 | 3.22 | 3.89 | 2.98 | 5.06 |
|  | K_2_O (%) | 3.14 | 3.08 | 3.1 | 2.88 | 1.47 | 1.55 | 2.28 | 1.55 | 3.64 |
|  | P_2_O_5_ (%) | 0.2 | 0.2 | 0.38 | 0.26 | 0.21 | 0.19 | 0.39 | 0.19 | 1.92 |
|  | SO_3_ (%) | 0.05 | 0.01 | 0.04 | 0.06 | 0.17 | 0.4 | 0.22 | 0.15 | 0.24 |
|  | Cl (ppm) | 66.03 | 95.33 | 123.25 | 395.06 | 5463 | 13164.28 | 6528.61 | 2962.22 | 0.22 |

**Table S4** **Akaike weights calculated from local-scale PLN- and NB-fitted SADs.** Where NA indicates that the fitting procedure did not converge, which is usual for small datasets.

|  | **Dataset** | **wPLN** | **wNB** |
| --- | --- | --- | --- |
| **Bacterial 16S** | MP | 1.000 | 0.000 |
|  | BP | 1.000 | <0.001 |
|  | RR | 1.000 | <0.001 |
|  | HI | 1.000 | <0.001 |
|  | AF | 1.000 | <0.001 |
|  | OW | 1.000 | <0.001 |
|  | RL | 1.000 | <0.001 |
|  | HV | 1.000 | <0.001 |
|  | TR | 1.000 | <0.001 |
| **Eukaryotic 18S** | MP | 1.000 | 0.000 |
|  | BP | 0.153 | 0.847 |
|  | RR | 1.000 | <0.001 |
|  | HI | 0.917 | 0.0830 |
|  | AF | 0.417 | 0.583 |
|  | OW | NA | NA |
|  | RL | NA | NA |
|  | HV | NA | NA |
|  | TR | <0.001 | 1.000 |
| **Archaeal 16S** | MP | 0.387 | 0.613 |
|  | BP | NA | NA |
|  | RR | 1.000 | <0.001 |
|  | HI | 1.000 | <0.001 |
|  | AF | 1.000 | <0.001 |
|  | OW | 1.000 | <0.001 |
|  | RL | 1.000 | <0.001 |
|  | HV | 0.959 | 0.0405 |
